# Experiences of hospitalization among pregnant women with preterm labor in Korea: a phenomenological study

**DOI:** 10.4069/kjwhn.2021.09.09

**Published:** 2021-09-30

**Authors:** Joon-Young Lee, Yeoungsuk Song

**Affiliations:** 1 College of Nursing, Daekyeung University, Gyeongsan, Korea; 2 College of Nursing, Kyungpook University, Daegu, Korea

**Keywords:** Emotions, Hospitalization, Perception, Premature obstetric labor, Qualitative research

## Abstract

**Purpose:**

The purpose of this study was to describe pregnant women’s lived experiences of hospitalization due to preterm labor in Korea.

**Methods:**

This qualitative study adopted a phenomenological approach. Individual in-depth interviews were conducted with nine participants, over the age of 20 years, who had been hospitalized for more than 1 week after being diagnosed with preterm labor. All interviews were audio-taped and verbatim transcripts were made for analysis. The data were analyzed following Colaizzi’s phenomenological method.

**Results:**

The participants’ ages ranged from 26 to 36 years, and all were married women. They were hospitalized for 13.1 days on average. Five thematic clusters emerged from the analysis. ‘Withstanding hospitalization for the fetus’s well-being’ describes women’s feelings during preterm labor and their endurance during their prolonged hospitalization, rooted in their conviction that the fetus comes first. ‘Endless frustration in the hospital’ encompasses women’s emotions while lying in bed and quietly thinking to themselves. ‘Unmet physiological needs’ describes participants’ awareness of their inability to independently handle human physiological needs given the need for careful and limited movement. ‘Gratitude for the support around oneself’ reflects the support from family and medical staff. ‘Shifting perceptions and accepting one’s circumstances’ describes accepting hospitalization and making efforts to spend their remaining time in the hospital in a meaningful way.

**Conclusion:**

The findings in this study provide a deeper understanding and insights into the experiences of Korean women with preterm labor during hospitalization, underscoring the need to develop interventions for these patients.

## Introduction

The total fertility rate in Organisation for Economic Cooperation and Development countries is 1.4 to 1.9 per woman on average, whereas South Korea (hereafter, Korea) recorded a rate of less than 1 per woman in 2018, which was the lowest in the world [1]. Korea’s total fertility rate in 2019 was 0.92, indicating that it has continued to decrease every year. This decrease in the total fertility rate has been accompanied by an increasing proportion of premature births (7.6% in 2017 and 8.1% in 2019). This proportion is now 1.4 times higher than it was 10 years ago [2].

Preterm birth causes various complications, including organ immaturity, neurological damage, disability, and developmental delay in newborns [3,4]. Preterm labor is the most common cause of premature birth, and uterine contractions begin before 37 weeks of pregnancy with progressive changes such as cervical dilatation and effacement [5]. Since preterm labor substantially increases the risk of premature birth, it is emerging as a very important obstetric problem [5,6]. According to the statistics of the National Health Insurance Corporation of Korea, 35,542 pregnant women received inpatient treatment for preterm labor in 2015; this number grew to 44,282 in 2016 and has subsequently remained above 40,000 (e.g., 43,178 in 2019) [7]. Since premature birth due to preterm labor is highly preventable [8], high-quality nursing care should be provided while pregnant women with preterm labor are hospitalized and treated. In the context of the national crisis of the low birthrate, it is very important to improve the quality of prenatal pregnancy management in order to overcome and cope with the gradually increasing decrease in the fertility rate and the increase in the premature birth rate and premature infant mortality [9]. Therefore, in order to improve the health of pregnant women and newborns, specific prenatal nursing interventions for pregnant women with preterm labor should be prepared.

Pregnant women diagnosed with preterm labor are hospitalized to maintain pregnancy. During hospitalization, they are placed on bed rest, have regular testing to monitor their symptoms, and receive pharmacological treatment with uterine contraction inhibitors [6]. Bed rest is a standard component of treatment for pregnant women with preterm, as it reduces intrauterine pressure by limiting physical activity and increases intrauterine blood flow to promote growth of the fetus in the uterus, thereby reducing the risk of premature delivery [10].

Bed rest is essential for pregnant women with preterm labor, but they experience physical discomfort due to the strict limitations on their daily activities [11,12]. In addition, because preterm labor requires long-term hospitalization, pregnant women can perceive it as a crisis situation [6]. In addition to the financial burden of long-term hospitalization, pregnant women experience social difficulties (e.g., separation from family) and feel psychological anxiety due to uncertainty about their own condition and the safety of the fetus. While coping with these problems, they experience various forms of stress [6,11]. Therefore, psychological support and individualized nursing interventions are needed. However, the nursing care provided for pregnant women with preterm labor has been limited to pharmacological interventions with uterine contraction inhibitors and bed rest, focusing only on maintaining pregnancy [6]. Thus, there is a need for a qualitative study to provide comprehensive insights into the actual experiences of pregnant women with preterm labor by understanding, describing, and explaining the meaning of their hospitalization experiences from hospitalization to delivery.

Phenomenological research is suitable for the purpose of this study, since it is an approach to obtain a vivid understanding of experiences by focusing on actual events experienced by research participants in context [13]. In addition, Colaizzi’s analysis method focuses on discovering and stating common characteristics that emerge from the study subjects’ descriptions of their feelings and experiences [14].

The purpose of this study is to promote an understanding of the hospitalization experiences of pregnant women with preterm labor by grasping the meaning of those experiences in a holistic, in-depth manner. The research question of this study was ‘What is the hospitalization experience of pregnant women with preterm labor?’ The results of this study can be used as basic data for the development of practical nursing interventions so that pregnant women with preterm labor can progress through hospitalization to have a healthy delivery.

## Methods

Ethics statement: This study was approved by the Institutional Review Board of Kyungpook National University (2019-0124). Informed consent was obtained from the participants.

### Study design

This phenomenological qualitative study was conducted to understand the overall meaning of the hospitalization experiences of pregnant women with preterm labor in Korea. This study was prepared according to the COREQ (Consolidated Criteria for Reporting Qualitative Studies) guidelines (https://www.equator-network.org/reporting-guidelines/coreq/additional references).

### Participant selection

The participants were purposively sampled according to the following criteria: pregnant women aged 20 years or older who received uterine relaxants and bed rest after being diagnosed with preterm labor at a tertiary university hospital in Daegu, Korea, and had been hospitalized for more than 1 week. A total of nine people participated, and recruitment and interviews continued until data saturation was confirmed.

Pregnant women who met the inclusion criteria were introduced through the attending physician and nurse, and a researcher directly visited those who indicated interest. The purpose of the study was explained and they could choose to participate voluntarily. Separate consent was obtained for recording the interviews.

### Data collection and setting

In-depth interviews were conducted based on semi-structured questions from August 21, 2019 to March 31, 2020. The researcher checked each participant’s treatment and examination schedule on the day of the interview and approached at a quiet time when there was no schedule conflict and checked with the participant whether it would be possible to conduct the interview. After obtaining voluntary written consent, we moved to a quiet room to protect the participant’s privacy and have a more in-depth conversation while maintaining a supine position. If the participant was in a single room, the interview was conducted in the hospital room. No other person was present during the interview. The interviewer sat in a chair next to the bed and made eye contact with the study participant, paying attention to meaningful verbal expressions and nonverbal expressions such as tone, behavior, and facial expressions, and recorded them in the field notes. The main question of the interview was ‘what was your experience after being hospitalized upon being diagnosed with preterm labor?’; and the sub-questions were ‘what difficulties did you experience while being hospitalized?’ and ‘how did you solve the difficulties caused by hospitalization?’ The in-depth interviews ranged from 40 to 90 minutes. The interviewer also used field notes and recorded the interview after obtaining prior consent from the participants. One interview was conducted per day, and a small gift was provided to the study participants after the interview was completed. Transcription was done immediately and three out of the nine participants were contacted by phone (up to three times) to confirm or supplement ambiguous content. The additional interviews took between 20 and 30 minutes. At the end of one interview, meaningful expressions were selected based on the transcripts of the interview, after which the next interview was conducted. Data saturation was reached when no new topics appeared when analyzing the interview data.

### Qualitative data analysis

The data transcribed in this study were analyzed according to Colaizzi’s seven-step process [14]. In the first step, the meaning of the interview was explored by repeatedly reading and underlining the transcript while recalling the circumstances of the interview on the day of the interview. Then meaningful statements were selected. In the third step, in order to clearly discover what participants wanted to reveal, we went back to the original data to uncover hidden meanings and describe them in a general form. Next, the meanings were organized into categories and compared with the supporting data to identify any contradictions or awkwardness. The consistency of the attributes and levels of each category was checked and if it was not valid, we returned to the original data and reexamined the meaning of the statements. In step 5, the theme clusters and themes were comprehensively described. The long-term hospitalization experiences of pregnant women with preterm labor were completely described in step 6, by integrating the theme clusters and thematic collections derived from participants’ statements about their experiences. Lastly, the long-term hospitalization experiences of pregnant women with preterm labor were clearly described and the structure of the meaning was described by presenting the original data of the study participants in detail, along with explanations of each topic and topic collection.

### Establishing research rigor

To ensure rigor of this study, the following efforts were made according to the evaluation criteria of Guba and Lincoln [15]. For credibility, participants who were able to sufficiently share their experiences of long-term hospitalization due to preterm labor were carefully chosen for interviews. The researcher also personally interviewed each participant, and verbal and nonverbal responses were recorded for accuracy. In addition, the researcher referred to internet community postings related to preterm labor to further verify that the participants’ stories were relevant, thereby upholding reliability. For transferability, two pregnant women with preterm labor who did not participate in the study were invited to read the study results in order to identify and support applicability of the study. Third, for dependability, Colaizzi’s data analysis procedure was presented and each theme and cluster was explained to support consistent and repeatable study findings. In addition, the findings were evaluated by a nursing scholar with extensive experience in qualitative research to ensure consistency. Lastly, the strategies used to secure reliability, transferability, and dependability, as mentioned above, can strengthen confirmability. We practiced bracketing to reduce prejudice as much as possible and sought neutrality in interpreting the data. Member checking was done with participants to enhance confirmability.

### Qualitative research preparation of the researchers

The interviewer was a woman nurse who had worked in the delivery room of a tertiary hospital for more than 2 years, and thus had knowledge and experience about pregnant women with preterm labor and was able to establish rapport with pregnant women. She has stayed informed of the latest trends in prenatal interventions, including preterm labor, and has given poster presentations on the topic of quality management and improvement for pregnant women admitted to the delivery room, as well as participating in related conferences. The researchers completed qualitative research classes in graduate school and participated in academic conferences and workshops to study qualitative research in-depth and to become better qualified as qualitative researchers.

### Relationship with participants

The researchers did not know the participants in advance, and consent was obtained through a pre-interview. Participants were informed that the researchers were nurses and understood the purpose of the study.

## Results

### Characteristics of participants

The nine participants’ age ranged from 26 to 36 years, all were married, and their average length of hospitalization was 13.1 days. In addition to preterm labor, additional diagnoses included gestational diabetes, placenta previa, and cervical incompetence. Six of the nine participants were primiparous women (Table 1).

### Major theme clusters

Through an analysis of the data collected through in-depth interviews based on the research questions, five theme clusters, 13 themes, and 47 meaning units were derived (Table 2). The analyzed theme clusters were ‘withstanding hospitalization for the fetus’s well-being,’ ‘endless frustration in the hospital,’ ‘unmet physiological needs,’ ‘gratitude for the support around oneself,’ and ‘shifting perceptions and accepting one’s circumstances.’

### Theme cluster 1: Withstanding hospitalization for the fetus’s well-being

This topic collection is about pregnant women’s feelings and perceptions of hospitalization as the hospitalization period was prolonged due to preterm labor. As the hospitalization continued, the difficulties faced by pregnant women increased day by day. In addition to physical difficulties, they felt the burden of treatment cost and continuous worries about the children left at home and the household chores. Despite these difficulties, however, they were able to endure the hospitalization because of their conviction that the fetus came first. This collection of themes includes ‘tolerating the pain and side effects of bed rest,’ ‘enduring difficulties due to treatment,’ and ‘feeling regret due to separation from children.’

#### Tolerating the pain and side effects of bed rest

Bed rest was essential for the participants, to the point that routine daily activities were restricted. This significant decrease in participants’ activity level caused indigestion, weight change, loss of muscle mass, muscle weakness, and lethargy. Although these physical changes were painful, participants believed they had to endure unconditionally to give birth to a healthy child, as seen in the following statements:

*I’m usually skinny, but I lost more weight because I couldn’t walk at all, not even after eating, so I can’t digest well, so I can’t eat well. I’m trying to eat a little bit because of my baby. My baby keeps growing but I’m losing weight, maybe because of muscle loss, so I wonder what strength I’ll have left to push in labor… but there is nothing I can do right now. I’m just trying to hold out as much as possible for now. (Participant 4)*
*As the weeks go by, my belly is growing a lot, my back hurts, I’m out of breath and sometimes it’s even more uncomfortable when I lie down. Since I am not active, my digestion is not working, so my stomach juices come up at night. Before I was hospitalized, my physical strength was good, but after being hospitalized and starting to lie down all the time, my physical strength seems to be dropping even more. (Participant 8)*

#### Enduring difficulties caused by treatment

Participants regularly received uterine contraction inhibitor administration, uterine contraction tests, cervical length examinations, and ultrasound examinations. Despite the side effects of these medications, such as tachycardia and hand tremor, and back pain due to testing for uterine contractions, which required them to lie for 20 minutes in one position, they were willing to maintain and endure the treatment. Some expressed embarrassment during measurements of the length of the cervix. In addition, participants were concerned about hospitalization and treatment costs, but said that the fetus came first. The following are some significant statements:

*I was using the machine (toco transducer) to watch the contractions, but it felt like my back was breaking. Then medication was started but the side effects of the uterine contraction inhibitors were severe, my pulse rate and heart palpitations were so severe that my hands were shaking. So it made me very uncomfortable. At the same time, while monitoring uterine contractions, the drug dose had to be increased, so it was very difficult. Even though it was hard, I had to endure it anyway because of my baby ... And because it was best to try to get as close to term as possible, I endured and continued with the treatment. (Participant 2)*
*When I first started the medicine, my heart was trembling, the back of my head was pounding, my shoulders felt cramped, and my head hurt. However, I have no choice but to use this drug. Because of the side effects, I took another drug (Trectosil) up to four times. I’m worried about it because insurance only covers up to the third use. But rather than focusing on that, I just hope my baby can endure it. (Participant 6)*

#### Feeling regret due to separation from children

Among participants with children at home, although they tried to relieve their longing through video calls, they still worried about the child(ren)’s well-being, repeatedly expressing that they wished to see them. For the sake of the fetus, however, they bravely faced this inevitable parting, as seen in the following excerpts:

*My mother-in-law is looking after the children, and I heard they’re having a hard time because I am not there. They can’t see their mother’s face, so they keep looking for me and calling for me. They’re crying every day while at her house. The kids are 6 and 4 and they’ve never been apart from me (crying). When we do video calls they say ‘Mom, come quickly’ and I can hear them crying. I’m in tears too. Still, I can’t cry, so I hold on tight (tears). But (touching her stomach) I have no choice but to be hospitalized for the baby. (Participant 3)*
*One phone call came with the child crying (tears). I had to be hospitalized unexpectedly, so I couldn’t explain to my child properly. That still haunts me. My parents are watching my child, but I feel bad when he cries while we do video calls. We have visiting time but I don’t want to see my child because I think my heart will ache even more if we meet. (Participant 6)*

### Theme cluster 2: Endless frustration in the hospital

This collection of themes relates to the emotions that participants experienced while maintaining a lying position and thinking quietly. Participants fell into a morass of thoughts and became depressed as they tried to find the cause of preterm labor. Looking out the window of the hospital room, they longed for discharge and going home. This theme set includes ‘self-blame for having preterm labor,’ ‘disconnection from the outside,’ and ‘unattainable discharge.’

#### Self-blame for having preterm labor

The participants compared themselves with normal pregnant women and thought that the reason they had preterm labor was because they were not careful about their bodies after getting pregnant or had somehow triggered preterm labor. In addition, regrets for the past and self-blame were seen in the following sample statements:

*At first, I was hospitalized for preterm labor and blamed my job for it. I wondered if it’s because I was standing all day, saying that what I’m doing is really hard, and if I had a strong womb, my child wouldn’t have to suffer like this. Because I have a very weak cervical neck, I don’t have the strength to support the child, so I think that uterine contractions are also occurring, and I keep blaming myself. (Participant 2)*
*Did I use my body too much after getting pregnant? Did I not manage it well? As a mother, I felt things like guilt. I thought I should have been more careful. Because I already have a child requiring care and I was also working. I was just carrying things. Maybe if I had been more careful, maybe this wouldn’t have happened. I regret this situation. (Participant 6)*

#### Disconnection from the outside

During hospitalization, all outside activities were restricted due to bed rest. The curtain cut off interactions with others and the participants spent boring days lying in bed. This was very frustrating, and there were even cases where they tried to go out secretly, avoiding the eyes of the medical staff, as seen in the following:

*It’s so boring here. I want to go out for a little while. The most frustrating thing is not being able to go out. The visiting hours are limited, and the only thing I can see is the wall. When I go for an ultrasound, just looking out the window for a moment gives me a breather. (Participant 3)*

*Being here is so mentally exhausting. I don’t know if the sun is up or if it’s raining (sigh). It was so frustrating that I heard that there was a bazaar on the first floor of the hospital, so I secretly went to the first floor with my husband to get some refreshments. However, the nurse came around and saw me and scolded me (laughs). (Participant 7)*

#### Unattainable discharge

Participants were very eager to be discharged, but were confused about delays in their discharge and the unclear discharge plan. Participants were distressed because their hopes for discharge were shattered as the treatment plan was changed frequently according to their symptoms of uterine contractions. Some participants also expressed conflicting thoughts of wanting to hide symptoms from the medical staff in order to be discharged. Excerpts include the following:

*During the doctor’s rounds, I kept saying that I would like you to discharge me, but they just say no, and they don’t tell you exactly when discharge is possible, so it’s difficult. I don’t know how long I will have to stay here, so I think it’s too much (gets emotional). Should I really give up on getting discharged? It seems like all hope is lost when you think that you have to be here until the baby arrives. (Participant 7)*
*How long do I have to be here? I wonder how many days I will have to be hospitalized. Originally, I was supposed to go if the medications stopped the contractions but they didn’t stop the medication, maybe because I had some contractions the other day. I can probably go home after the medication is stopped but I don’t know when. Sometimes I wonder if I should put up with the preterm labor pains, because I’m afraid I won’t be able to go home if I say I feel contraction pains. (Participant 3)*

### Theme cluster 3: Unmet physiological needs

This collection of topics deals with conflicts related to basic human physiological needs, as participants realized that they could not perform many activities independently due to the need for extreme care in their movements, making it necessary for them to receive help from others. This topic set includes themes of ‘lack of self-hygiene measures’ and ‘limited defecation care.’

#### Lack of self-hygiene measures

Showering in a standing position was prohibited because of the risk of uterine sagging and the possibility of the child descending. Although they were aware they had to be careful because of preterm labor, they lamented their unclean appearance and expressed distress and depressed feelings, as seen in their statements:

*I have never taken a shower since I was hospitalized. When I asked for the needle to be removed to take a shower, the nurse said ‘you can’t shower here.’ So when can I wash? Yesterday, I cried because of it. I don’t want to give birth without a shower (sigh). Washing is really important for people, but it was the first time I realized how important it really is. Although the baby is important, I wish more consideration was given to us moms, in terms of cleanliness and hygiene management. Also, when I want to change clothes, I have to ask them to remove the needle, so there is nothing I can do (independently). Every time I do something, I have to get the nurse’s permission and help, one by one, so my quality of life has really deteriorated. (Participant 7)*

#### Limited defecation care

Participants had to lie in bed to defecate and urinate, and because they could not go to the toilet themselves, they asked caregivers, nurses, and guardians to bring a bedpan and to empty it. All the participants complained of difficulty in urinating and defecating while lying down, and also said that they did not dare push because of the risk of the fetus descending. This led to constipation in some participants. Yet, they were reluctant for others to see their feces and urine, and they viewed toilet management as another source of stress, as seen in the following excerpts:

*I’m lying down and being still, so there’s no activity, so I can’t have a proper bowel movement. There is a lot of stress that comes from not having a smooth bowel movement. There was one time when the stool got caught in between. If you try to force it the baby may come down and labor pains may start, so I couldn’t do anything. Eventually the intern ended up removing it and I know this is better than pushing, but it was too embarrassing. It’s not just this. You have to get permission from the nurse and ask if you can urinate. So, I couldn’t be free to urinate whenever I wanted to. There were always restrictions. (Participant 1)*
*I barely urinate and I couldn’t pass stool for 5 days, but you really can’t try to do it while lying down. The attempt itself doesn’t work. There was a risk that the child would come down if any of the postures were too strained, so I couldn’t even sit on my own. And it’s too humiliating to call someone to empty the bedpan after you urinate because you can’t even manage it yourself. (Participant 4)*

### Theme cluster 4: Gratitude for the support around oneself

This theme shows the emotions participants felt toward family and the medical staff. They expressed gratitude and regret to their families and medical staff for being hospitalized for a long time. This collection of themes includes subthemes of ‘being sorry and thankful to family’ and ‘encouraged by medical staff.’

#### Being sorry and thankful to family

Participants foremost thanked their families for their support. They also felt sorry that their husbands, family, parents-in-law, and child(ren) suffered and worried while having to adjust their lifestyles due to participants’ hospitalization. Participants also expressed regret for not being able to properly do prenatal education (*taegyo*, in utero care for and bonding with the baby) mixed with gratitude for emotional support. Sample statements include the following:

*Because I’m having a really hard time, my family looks at me and worries whether I’ll be able to endure until it’s time to give birth. My parents were more worried about me than the children. My family also seems to accumulate a lot of anxiety and fatigue. There is also stress. So I’m always feeling sorry for my family but I’m grateful that I can rely on them. The anxiety I’ve been feeling for a long time would have been transmitted to my baby but I haven’t done prenatal education properly, so I’m sorry for my baby as well. (Participant 1)*
*Many family members are suffering a lot because of me. My in-laws are sacrificing to look after the children, and my husband visits every single day. He comes even it’s just for a moment, but I can imagine how difficult it is to come because he’s also working... So I am very grateful and really feel supported. My mother works, so she can’t come often but she calls me every day, so I’m thankful for her concern. She gives me a lot of support by saying that everything will be okay. (Participant 3)*

#### Encouraged by medical staff

Participants initially were unhappy with the medical staff for restricting their daily life and for being strict about treatment, thinking that the treatment was excessive and staff were oversensitive to the signs of preterm labor. However, as time passed, they understood that the staff were trying to protect the fetus and thus felt grateful and friendly to the kind medical staff who supported them emotionally.

*I really thank the medical staff for being so kind here. I think we’ve gotten to know each other a bit more now. The truth is, I used to say bad things about my doctor to my husband because I didn’t like his style. Because for my second child, when I had belly cramps and went to the emergency room, it was at a similar time of pregnancy but I didn’t need hospitalization. This time the cramps felt lighter and went away, so what’s the difference? I thought the hospital was being too sensitive, not letting me go home and telling me not to move. I was really upset with the doctor. But now that I know why they’re doing this, I can brush it off. (Participant 3)*
*At first, I thought that the staff here were too harsh. The doctor kept telling me to stay in the hospital and lie down, but I was very resentful. But the staff once said, “Mom, you can get through it. You’re doing well now.” (Participant 7)*

### Theme cluster 5: Shifting perceptions and accepting one’s circumstances

This theme cluster is about the participants accepting that discharge will not be easy, and trying to cultivate their mental health during the rest of their time in the hospital. Participants comforted each other by sharing openly with other hospitalized pregnant women. In addition, they tried to accept the current situation that was given to them, interpret the meaning of hospitalization in their own way, and live their daily lives happily until childbirth. This theme set includes ‘taking comfort in shared similar experiences,’ ‘gratitude for the opportunity to look back on oneself,’ and ‘mastering self-control over one’s time.’

#### Taking comfort in sharing similar experiences

Participants showed considerable dependence on other pregnant women who were hospitalized together. Because they shared the same situation, they were comforted by and sympathized with each other, despite the limitations of being supine.

*No matter how close my friends are, even if they’re pregnant, none of their words touched me. No matter what they say and I thank them, but my anxiety remains. When it was nighttime with these mothers and I spoke one word at a time, it really touched me. It was so comforting. Because they share the same pain. Even when you say the same thing, I feel it’s more sincere. In the end, it’s more comforting because I’m someone who has the same experience. (Participant 1)*
*I asked for the curtain (between the beds) to be opened and had a conversation for the first time (with another woman with preterm labor), and my frustration was relieved. I also clear my mind while communicating with the mothers next to me. (Participant 8)*

#### Gratitude for the opportunity to look back on oneself

Participants found positive meaning through hospitalization as they looked back on themselves during the long hospitalization and were grateful they were given time to rest fully. At first, it was difficult to maintain the supine position, but they tried to accept and overcome the limitations of hospitalization by finding positive meaning for themselves, as illustrated in the following excerpts:

*I’ve been working for over 10 years, but this is the first time I’ve been constantly lying down and even sleeping like this. Even though I’m lying down and on medication, I thought that this was a period for rest. It is a healing time with the thought that God is giving us a little rest to protect my baby, even in this way. It’s also a stress-reducing environment. I’ve been focusing on my work all this time, so I couldn’t fully care for my child and lived my life centered on work. It made me look back. So, I want to think of it as a precious time to spend with my baby before s/he is born. It is meaningful and the best time, a time of healing, a time of rest. (Participant 2)*
*The lengthy hospitalization allowed me to see life again and got me looking back because I am experiencing something I have never experienced in my life, if I hadn’t had this experience, I would have had some limits when looking at certain situations. My perspective has also broadened. It’s all acceptable. For my first child parenting felt difficult, but if this child is born I don’t think it will be difficult at all. I have this child who has endured this time well, so I don’t think parenting will be hard at all. This is the moment that really makes me feel reborn. In that sense, now is not the worst time. (Participant 9)*

#### Mastering self-control over one’s time

Participants learned how to spend each day in a comfortable and meaningful way in the hope of a healthy childbirth. Religious participants used their smartphones to look up videos and listen to religious books and music, or prayed quietly with a humble attitude, relying on the power of their faith. In addition, many participants spent much of their time enjoying themselves by watching movies, listening to music, and shopping for baby products.
*I spend most of my time lying down, looking at my phone, shopping for clothes for my baby on the internet, looking at baby products, and thinking about what I should do when my baby is born. If I wear earphones and watch movies or dramas occasionally and listen to my favorite music, the time passes quite well. (Participant 3)*
*I’m religious, and I start and spend the day listening to religious teachings or music on my mobile phone. (Being in the hospital) is boring, but I’m trying to have a good time until childbirth by watching movies on my phone, reading good articles, and making video calls frequently. If you do that, another day will pass, and my baby will spend another day in the womb. (Participant 9)*

## Discussion

This phenomenological study was conducted to understand the in-depth meaning of the vivid experiences of pregnant women who were hospitalized due to preterm labor. The five theme clusters are discussed below.

**Theme cluster 1:** ‘Withstanding hospitalization for the fetus’s well-being.’ Korean society commonly views motherhood as an instinctive role, framed in a culture that emphasizes responsibility and excellence in motherhood [16]. As such, the emphasis on the maternal role and responsibilities can further intensify the burden of pregnancy [17]. In high-risk conditions such as preterm labor, if the main focus centers on medical management and compliance with medical instructions, pregnant women may have negative perceptions of their situation and feel it to be burdensome. As such, a person-focused approach is all the more important [18].

Therefore, health care professionals should be mindful of the difficulties of preterm labor experienced by pregnant women, and thus provide strong emotional support to such pregnant women with as much attention as is given to therapeutic interventions. In this study, although women were struggling with the various difficulties they faced, the health of their unborn child was their foremost priority. In particular, women with children at home were very upset about not being able to be with them, but pulled themselves together to withstand the hospitalization for the health of the fetus. Korean women’s strong sense of responsibility and sacrifice for their children may cause them to feel guilty if they are unable to fulfill the role of caring and nurturing [19]. However, since maternal role confidence has been reported to improve the stress of preterm labor and enhance fetal attachment [20], developing a nursing intervention program that can enhance maternal confidence in pregnant women in preterm labor will be beneficial.

**Theme cluster 2:** ‘Endless frustration in the hospital.’ In most cases, the exact cause of preterm labor is unknown [21] and the uncertainty that patients experience in the treatment process hinders psychological stability [22]. This suggests that health care providers can reduce uncertainty by providing patients with sufficient explanations and information about the treatment process. Patients have high demands and expectations for informational support from health care providers about their condition and feel relief and satisfaction if they perceive that professional help has been extended [18]. Therefore, careful explanations about treatments, examinations, and the patient’s current status compared to before can provide important support for women with preterm labor. Nurses are in a good position both to provide timely information by cultivating their communication skills and to manage the emotions experienced by pregnant women during extended hospitalization.

**Theme cluster 3:** ‘Unmet physiological needs.’ Because participants’ daily activities were restricted, all activities were controlled, including going to the bathroom and showering. Greater physical discomfort experienced by pregnant women with preterm labor has been reported to be associated with a more negative emotional state [12]. Therefore, the difficulties that participants faced with basic hygiene and defecation were a major source of stress during hospitalization and adversely affected both their physical and psychological health. Considering a previous study [23] noting that higher stress increased nursing requirements in pregnant women with preterm labor, it is necessary to identify their stressors and provide adequate care. Bed rest is essential for pregnant women with preterm labor [11], but physical discomfort caused stress in this study, which has also been reported in the literature [12]. Therefore, it is necessary for health care providers to listen to pregnant women’s experiences to understand their discomfort and improve treatment measures so that they do not infringe on women’s basic needs [18]. Differentiated individual guidelines allowing partial hygiene management and defecation within a range that does not worsen preterm labor symptoms would be helpful.

**Theme cluster 4:** ‘Gratitude for the support around oneself.’ Although the participants had feelings of gratitude and regret toward their families, the emotional support from their families was a powerful force helping them to overcome their difficulties and they experienced a sense of unity in the family. In a study of 185 pregnant women in Korea, a higher degree of family support perceived by pregnant women was associated with lower stress [24]. Therefore, it is necessary for nurses to provide collaborative education to help patients’ families perform their role as a support system well and positively assist pregnant women when they are hospitalized for preterm labor. In addition, this study found that participants felt resentment and distrust of the strict treatment from health care providers, but expressed warmth and gratitude when they realized the true intentions and goals of treatment. Korean pregnant women hospitalized for preterm labor have been shown to have care needs requiring communication about the nature of preterm labor, medication, and sleep [25]. In addition, pregnant women with preterm labor mainly used emotion-focused coping during hospitalization, and educational and emotional nursing needs were higher than physical nursing needs [23]. Communication based on empathy and emotional support can provide emotional stability and comfort to the patient, which in turn, can impact the treatment process and outcome [18]. Therefore, health care providers should be mindful of the emotional status of pregnant women with preterm labor and strive to provide tailored informational support based on empathetic and therapeutic communication.

**Theme cluster 5:** ‘Shifting perception and accepting one’s circumstances.’ Pregnant women with preterm labor found strength in sharing their own experiences, which were unlike those of normal pregnancy, and they processed their accumulated emotions while comforting each other. A self-help group intervention was reported to be effective in relieving negative emotions for pregnant women on bed rest [26]. Nurses can create and guide self-help groups for pregnant women with preterm labor within the hospital to facilitate such sharing. This study also found that participants spent much time using smartphones as a way to overcome the dreary hospitalization period until childbirth, which aligns with a study of 613 people in 24 countries that found that pregnant women with preterm labor used the internet to relieve their psychological anxiety and obtain related information [27]. However, as higher smartphone dependency in pregnant women with preterm labor was associated with lower sleep quality [28], education and support for appropriate use of smartphones may also be beneficial.

### Limitations

This phenomenological study explored the hospitalized experience of pregnant women with preterm labor in Korea. Although two pregnant women with preterm labor who did not participate in the study were invited to read the study results, in order to identify and support applicability, the fact that our participants were only recruited from one institution may limit the transferability of the findings. As the number of multiparous women was limited, a follow-up study focusing on women with preterm labor who have children at home may be required for a further understanding of women’s experiences depending on their context.

Based on the results of this study, nurses can provide more sensitive care and consider supporting programs that facilitate self-processing and alleviate stress. We propose developing emotional support and individual counseling program that can be implemented in the hospital room and conducting a study to verify its effectiveness for minimizing stress while on bed rest for women with preterm labor during hospitalization. In addition, given that pregnant women with preterm labor are particularly concerned about their families while they are hospitalized, but they receive the greatest support from their families, family support education should be provided for the families of pregnant women who need to be hospitalized for preterm labor to increase the comfort of their hospital stay.

## Figures and Tables

**Table 1.** General characteristics of the participants (N=9)

No.	Age (year)	Hospitalization day	Gestational week	Cervical length (cm)	Diagnosis	Baby’s birth order
1	35	17	21.3	1.5	PTL, IIOC	1
2	36	14	32.0	1.8	PTL, IIOC	2
3	30	8	24.2	2.0	PTL, IIOC	2
4	26	10	31.0	2.3	PTL, IIOC	1
5	32	15	35.2	4.0	PTL, GDM	1
6	32	15	33.2	2.5	PTL, IIOC	3
7	29	10	34.2	4.0	PTL	1
8	28	14	33.1	3.5	PTL, placenta previa	1
9	31	15	33.3	3.3	PTL, GDM	1

GDM: Gestational diabetes mellitus; IIOC: incompetent internal os of cervix; PTL: preterm labor.

**Table 2.** Experiences of hospitalization of pregnant women during preterm labor

Theme cluster	Theme
Withstanding hospitalization for the fetus’s well-being	Tolerating the pain and side effects of bed rest
	Enduring difficulties caused by treatment
	Feeling regret due to separation from children
Endless frustration in the hospital	Self-blame for having preterm labor
	Disconnection from the outside
	Unattainable discharge
Unmet physiological needs	Lack of self-hygiene measures
	Limited defecation care
Gratitude for the support around oneself	Being sorry and thankful to family
	Encouraged by medical staff
Shifting perceptions and accepting one’s circumstances	Taking comfort in sharing similar experiences
	Gratitude for the opportunity to look back on oneself
	Mastering self-control over one’s time
